# Hypertonic saline in non–cystic fibrosis bronchiectasis (Hyper-BRONCHI): an updated systematic review and meta-analysis

**DOI:** 10.1186/s12890-026-04176-4

**Published:** 2026-02-12

**Authors:** Nhan Nguyen, Yacin Zawam, Nghi Bao Tran, Nathalia Alves de Barros e Lyra, Vinh Quang Tri Ho, David Downes, Vy Ngoc Dan Nguyen, Ha Duc Thien Le, Jafar Aljazeeri

**Affiliations:** 1 Faculty of Medicine, University of Debrecen, Egyetem ter 1, Debrecen, 4032 Hungary; 2 Faculdade Pernambucana de Saude, Recife, Pernambuco, Brazil; 3 Southern Ohio Medical Center, Portsmouth, Ohio USA; 4 Department of Rural Medicine, The University of New England, Armidale, Australia; 5 Department of Nursing, The University of Melbourne, Melbourne, Australia; 6 Medical City Hospital for Military and Security Services, Muscat, Oman; 7 University of Pittsburgh Medical Center, Pittsburgh, USA; 8 Drexel University College of Medicine, Philadelphia, USA

**Keywords:** FEV1, FVC, Hypertonic saline, Isotonic saline, Non-cystic bronchiectasis

## Abstract

**Background:**

Airway clearance is essential in managing bronchiectasis, yet the benefit of inhaled hypertonic saline (HS) in non–cystic fibrosis (non-CF) disease remains unclear. While HS improves mucociliary clearance and clinical outcomes in cystic fibrosis, supporting evidence in non-CF bronchiectasis has been limited. With newly available trial data, this updated meta-analysis reassesses the efficacy of inhaled HS in adults with non-CF bronchiectasis.

**Method:**

We systematically searched PubMed, Cochrane CENTRAL, and Embase for randomized controlled trials (RCTs) comparing inhaled HS with control group (Non-HS), either isotonic saline or standard of care, in adults with non-CF bronchiectasis. Outcomes included changes in forced expiratory volume in one second (FEV₁), forced vital capacity (FVC), and pulmonary exacerbation frequency within 52 weeks. The risk of bias was assessed with the Cochrane tool. A random-effects model was applied.

**Results:**

Four RCTs involving 386 adults were included. Compared with Non-HS, HS did not significantly improve FEV₁ (SMD 0.03; 95% CI − 0.07 to 0.13; low certainty) or FVC (SMD 0.10; 95% CI − 0.06 to 0.25; low certainty), and pulmonary exacerbation rates (SMD − 0.02; 95% CI − 0.48 to 0.45; very low certainty).

**Conclusion:**

In adults with non-CF bronchiectasis, inhaled HS offers no meaningful advantage over Non-HS for lung function or exacerbation reduction. Larger, high-quality RCTs are needed to identify potential benefits.

**Supplementary Information:**

The online version contains supplementary material available at 10.1186/s12890-026-04176-4.

## Key points

Question: Does inhaled hypertonic saline (HS) provide better lung function outcomes for patients with non-cystic fibrosis bronchiectasis (non-CF bronchiectasis) ?

Finding: Overall, hypertonic saline (HS) yielded outcomes comparable to those of the non-HS group (including isotonic saline or standard of care) with respect to FEV₁, FVC, and the annual number of pulmonary exacerbations. These findings were consistent during long-term follow-up of up to 52 weeks.

Meaning: Up to date, the data on HS inhaled therapy for non-CF bronchiectasis is limited in high-quality studies, highlighting the need for further non-CF bronchiectasis-specific RCTs, focusing on HS of NaCl 6–7%.

**Supplementary Information:**

The online version contains supplementary material available at 10.1186/s12890-026-04176-4.

## Background

Airway clearance remains a cornerstone of bronchiectasis management [1], yet the clinical benefit of inhaled hypertonic saline (HS) in non–cystic fibrosis (non-CF) disease remains uncertain. Although HS has been shown to improve mucociliary clearance and clinical outcomes in cystic fibrosis (CF), evidence supporting its use in non-CF bronchiectasis has been limited [1, 2]. Historically, three randomized trials [3–5] evaluated HS in non-CF bronchiectasis and reported improvements in FEV₁ and FVC; however, these studies were limited by small sample sizes, potential selection bias, and modest dropout rates. Two prior meta-analyses [6, 7] attempted to synthesize the available evidence: one suggested a possible benefit of HS on pulmonary function [6], while the other did not perform a pooled analysis and instead relied on individual trial results [7]. Interpretation of both reviews was restricted by the small number of eligible trials (fewer than three), heterogeneity in outcome measures, and the inclusion of at least one study at high risk of bias.

Recently, the CLEAR trial [8], an open-label, two-by-two factorial randomized study conducted across 20 sites in the United Kingdom, reported no significant differences between HS and control groups in pulmonary outcomes, including the mean number of pulmonary exacerbations over one year. Notably, the CLEAR trial [8] had a substantially larger sample size, enrolling 288 patients with non–cystic fibrosis bronchiectasis, compared with fewer than 100 patients in total (*n* = 98) across the three earlier RCTs [3–5]. Consequently, this study contributed greater statistical weight to the pooled estimates. In addition, the CLEAR trial [8] provided data on the mean number of pulmonary exacerbations over a follow-up period extending to 52 weeks, enabling a more robust evaluation of the long-term effects of hypertonic saline in non–CF bronchiectasis. Incorporating these newly available data, we conducted an updated meta-analysis to reassess the efficacy of inhaled HS in adults with non-CF bronchiectasis, with a particular focus on lung function outcomes.

## Method and study designs

This systematic review and meta-analysis, Hyper-BRONCHI (PROSPERO ID: CRD420251238483), was conducted in accordance with the Cochrane Collaboration Handbook for Systematic Review of Interventions and reported following the PRISMA (Preferred Reporting Items for Systematic Reviews and Meta-Analysis) Statement guidelines [8, 9].

### Selection criteria and search strategy

A comprehensive search of major databases (PubMed, Embase, and Cochrane Central) was performed from inception to November 2025. The full search strategies for each database are provided in Supplementary. Reference lists of relevant systematic reviews and included studies were also manually screened for additional eligible trials. Study selection followed PRISMA guidelines and was performed in two stages. Titles and abstracts were independently screened by two reviewers (N.N. and Y.Z.) to identify potentially eligible studies. Disagreements were resolved by discussion or, when necessary, by consultation with a third reviewer (N.B.T.). Subsequently, the full texts of shortlisted articles were independently assessed for eligibility. Data extraction was performed independently and in duplicate by Y.Z., with N.A.d.B.e.L using a standardized form, with forest plot data double-checked by H.D.T.L. and V.N.D.N. Discrepancies were resolved through discussion or, if needed, by a third reviewer (N.N.). This meta-analysis was prospectively registered on PROSPERO (CRD420251238483) on November 23rd, 2025.

We searched for randomized controlled trials (RCTs) comparing inhaled HS with Non-HS control group, as defined by each trial, either isotonic saline [3–5] or standard of care [8], in adults with non-CF bronchiectasis. At the time of PROSPERO registration, the control group was defined as isotonic solutions, as these were the most commonly used comparators in prior studies. However, to ensure inclusivity and incorporate the CLEAR trial, we subsequently expanded the definition to include standard of care comparators beyond isotonic saline.

Eligible outcomes included changes in mean forced expiratory volume in 1 s (FEV₁), mean forced vital capacity (FVC), and mean pulmonary exacerbation frequency within 52 weeks. We excluded non-English publications; single-arm RCTs; observational studies; pediatric or CF-associated bronchiectasis trials; combination therapies without a standalone HS arm; conference abstracts; and subgroup analysis studies. For subgroup analysis, given that at least two RCTs reported the outcome, we aimed to evaluate long-term treatment duration (at least one year) and hypothesized a potential benefit of HS therapy over non-HS in patients with non-CF bronchiectasis. 

### Data synthesis and quality assessment

Data extraction followed predefined criteria. Three reviewers (N.B.T., N.A.d.B.e.L., and D.D) independently extracted baseline characteristics (Table 1) and outcome data. The certainty of evidence was assessed using the GRADE (Grading of Recommendations, Assessment, Development, and Evaluation) approach [10] by V.H.Q.T. and D.D., considering risk of bias, imprecision, inconsistency, indirectness, and publication bias. Outcomes were rated as high, moderate, low, or very low certainty. This evaluation informed the overall strength and reliability of the Hyper-BRONCHI meta-analysis findings.

**Table 1:** Baseline characteristics of included studies

Study	Design	Group	Age, mean (SD)	Sex (M/F)	BMI (kg/m²)	FVC % predicted (SD)	FEV₁ % predicted (SD)	Annual exacerbations	Key inclusion criteria
Kellett & Robert, 2011 [4]	Cross-over	—	56.6 (14.6)	14 / 14	NR	77.8 (23.4)	66.4 (26.1)	2.6	> 18 yrs; HRCT-confirmed bronchiectasis ≤ 4 years; stable chronic treatment allowed.
Nicolson et al., 2012 [5]	Parallel RCT	HTS	58 (15)	7 / 13	27.9	98.5 (17.8)	84.8 (20.5)	5.0 (median)	> 18 yrs; HRCT-confirmed bronchiectasis; clinically stable; daily sputum; ≥2 exacerbations/yr (past 2 yrs)
		IS	56 (15)	8 / 12	28.2	97.1 (18.0)	80.4 (21.1)	5.5 (median)	
Herrero-Cortina et al., 2018 [3]	Cross-over	—	64.0 (17.5)	10 / 18	24.3	NR	60.9 (24.6)	NR	> 18 yrs; HRCT-confirmed bronchiectasis; stable ≥ 4 weeks; sputum ≥ 10 g/24 h; able to perform ACT.
Bradley et al., 2025 [8]	Parallel RCT	HTS	65.3 (13.6)	60 / 86	28.1	NR	NR	3.4 ± 2.1	≥ 18 yrs; CT-confirmed bronchiectasis; ≥2 exacerbations/yr (or ≥ 1 post-COVID-19); daily sputum.
		No HTS	66.1 (12.3)	57 / 85	27.6	NR	NR	3.6 ± 2.1	

Data are presented as mean ± SD unless otherwise specified. “Median” indicates median (IQR)

*NR* Not reported, *HRCT* High-resolution computed tomography, *ACT* Airway clearance technique, *HTS* h=Hypertonic saline, *IS* Isotonic saline

Additionally, two independent reviewers (N.A.d.B.e.L. and V.H.Q.T.) assessed risk of bias using the Cochrane Risk of Bias 2.0 (RoB 2) tool [11], examining five domains: selection, performance, detection, attrition, and reporting bias. Each domain was rated as low, high, or unclear risk, with any discrepancies resolved by consensus or consultation with a third reviewer (N.N.).

All analyses were performed using random-effects models. Dichotomous outcomes were pooled using risk ratios (RRs) with 95% confidence intervals (CIs), while continuous outcomes were pooled using standardized mean differences (SMDs) to account for variations in lung function reporting units (e.g., FEV₁ or FVC % predicted versus liters). Pulmonary exacerbations were extracted as count-based outcomes, defined as the mean (or median) number of exacerbations per patient during the study follow-up period, as reported in the original trials. Due to inconsistent reporting formats across studies, such as the absence of standardization to patient-years or insufficient data to calculate rate ratios, pulmonary exacerbations were analyzed as continuous outcomes and pooled using SMDs, enabling synthesis across heterogeneous reporting methods. Statistical heterogeneity was assessed using the I² statistic and Cochrane’s Q test, with I² > 50% and *p* < 0.10 considered indicative of substantial heterogeneity. All analyses were conducted using RevMan version 5.4.

## Results

**Fig. 1:**
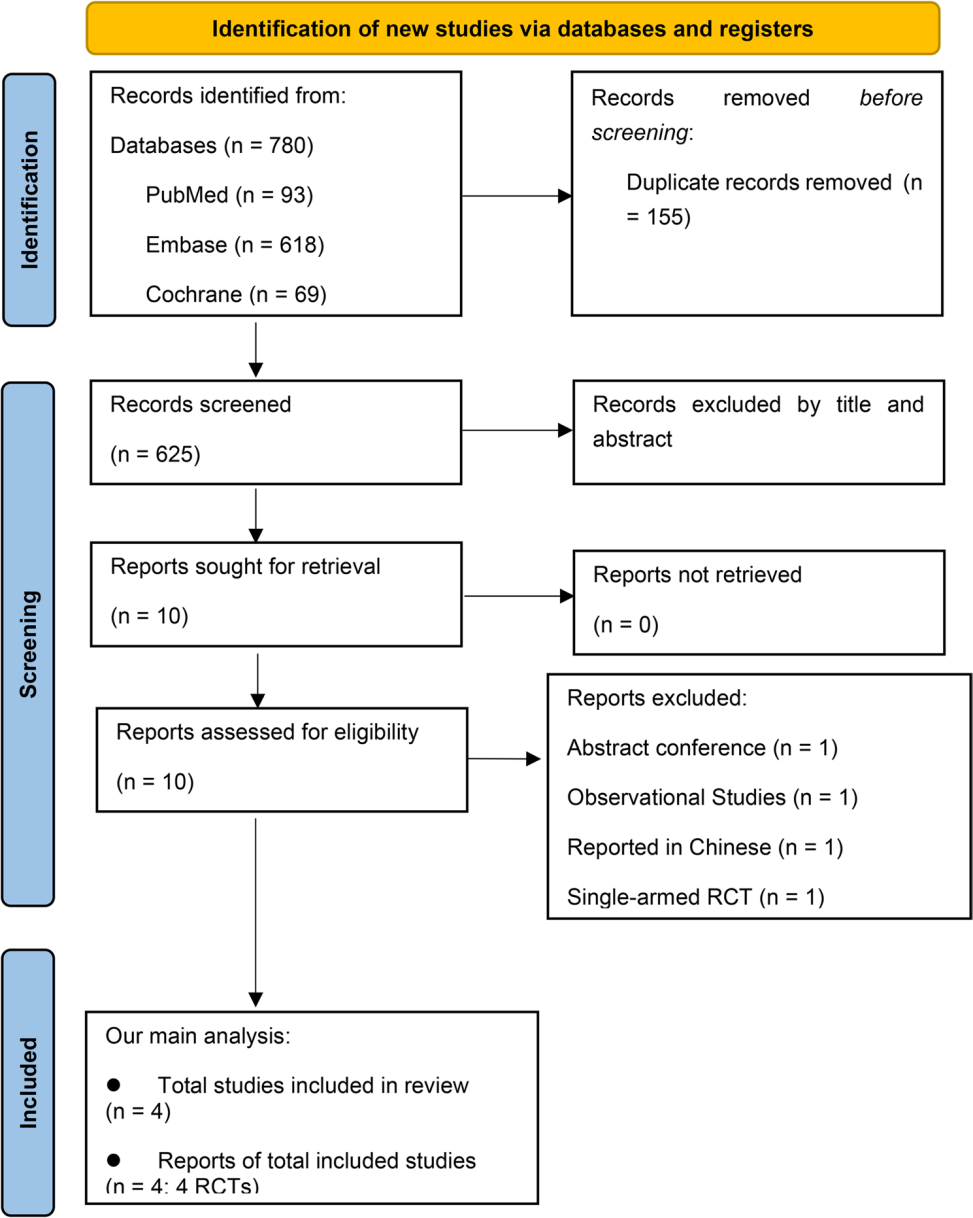
Flowchart of study selection. From: [9]

After screening 625 records and reviewing 10 full-text articles, four RCTs involving 386 patients met the inclusion criteria (Fig. 1). Participants across the trials were predominantly older adults (mean age ≥ 55 years), with a female predominance (> 55% in three studies [3, 5, 8]). Three trials reported BMI: two [5, 8] reported mean values > 27 kg/m², while one [3] reported a mean of approximately 24 kg/m². Baseline disease severity also varied: three trials [3–5] reported mean FEV₁% predicted values above 60%, whereas the CLEAR trial [8] used FACED scores, with a mean of approximately 2. Notably, CLEAR was the only trial that reported baseline smoking status. Further details regarding baseline characteristics of each trial are presented in Table 1.

**Fig. 2:**
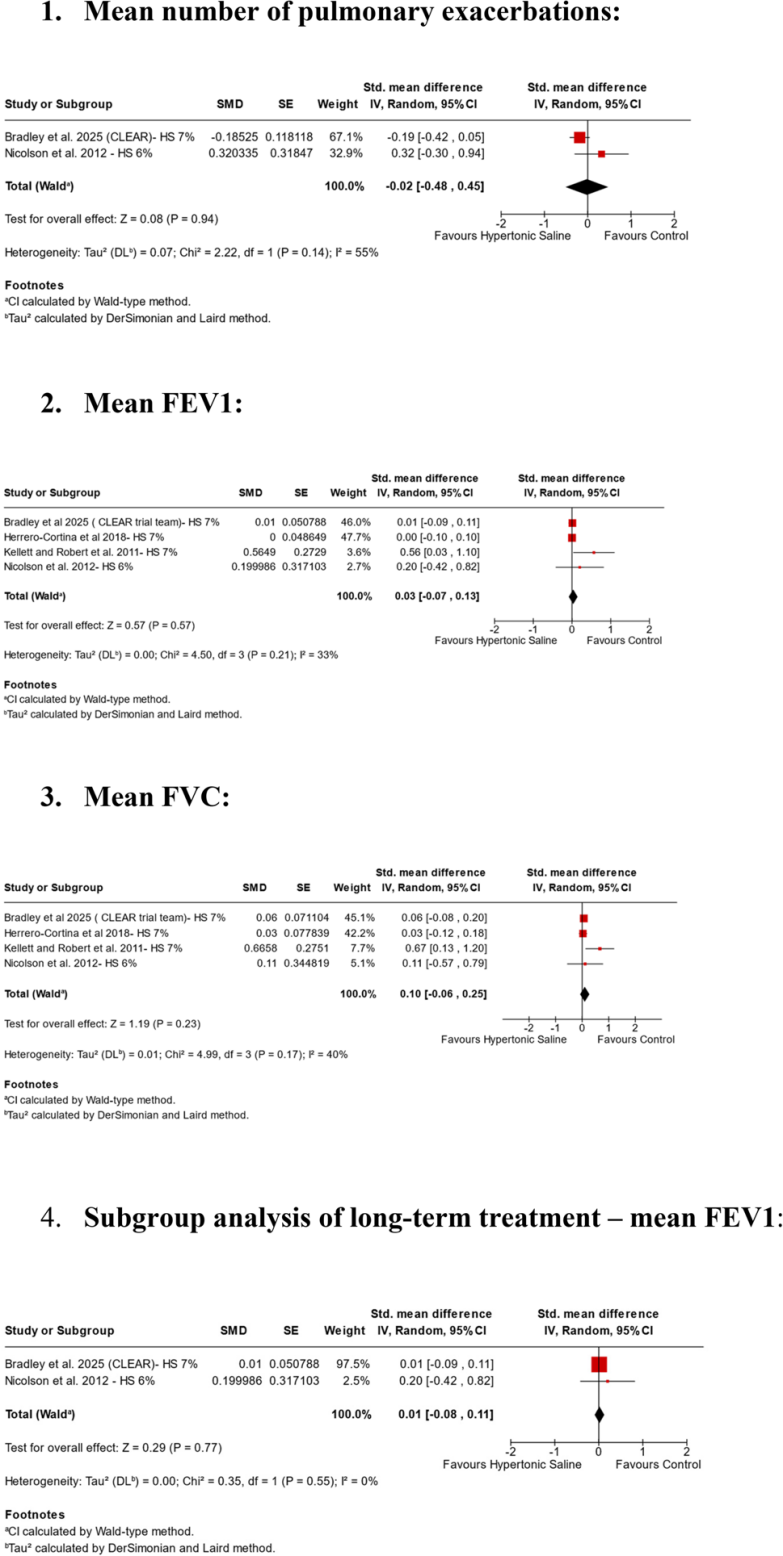
Pooled analysis of Hyper-BRONCHI

Compared with non-HS comparators, inhaled HS did not significantly improve FEV₁ (4 RCTs, SMD 0.03; 95% CI − 0.07 to 0.13; *p* = 0.57; I² = 33%; low certainty; Fig. 2), FVC (4 RCTs, SMD 0.10; 95% CI − 0.06 to 0.25; *p* = 0.23; I² = 40%; low certainty; Fig. 2), or annual pulmonary exacerbations (2 RCTs, SMD − 0.02; 95% CI − 0.48 to 0.45; *p* = 0.94; I² = 55%; very low certainty; Fig. 2). Statistical heterogeneity was moderate (I² >25% but < 40%). Two trials [3, 4] were judged to have a higher risk of bias, whereas the remaining two [5, 8] were assessed as low risk (Supplementary Table 2).

The trial by Kellett and Roberts [4] was identified as an outlier contributing to heterogeneity in the pooled analysis. It was the only study reporting substantial differences between HS and isotonic saline for both FEV₁ and FVC, and it was also the only trial judged to have a high risk of bias. This classification was based on key methodological limitations, including single blinding, inconsistent outcome measurement units, missing tolerability data, and limited reporting on allocation concealment and prespecified analyses. These factors may have introduced bias and likely contributed to the divergent conclusions observed in a previous meta-analysis [6]. Herrero-Cortina et al., [3] was rated as having “some concerns” for overall risk of bias, primarily due to missing outcome data resulting in an 18% dropout rate. The sensitivity analysis, conducted by sequentially removing individual trials to assess their influence on the overall results, yielded findings consistent with the primary analyses for mean FEV₁ (Supplementary Fig. 3.1–3.4) and mean FVC outcomes (Supplementary Fig. 4.1–4.4). However, sensitivity analyses for annual pulmonary exacerbations were limited due to the inclusion of only two trials.

A subgroup analysis restricted to long-term treatment (≥ 1 year), which included two trials [5, 8], yielded results consistent with the primary FEV₁ analysis and mitigated concerns regarding bias (2 RCTs, SMD 0.01; 95% CI − 0.08 to 0.11; *p* = 0.77; Fig. 2). Notably, both trials enrolled patients with frequent annual pulmonary exacerbations, with mean values of approximately 5 exacerbations per year in Nicolson et al. (2011) [5] and 3 per year in the CLEAR trial [8]. We were unable to confirm our initial hypothesis, possibly due to limited statistical power to detect clinically meaningful differences between therapies.

## Discussion

This meta-analysis (Hyper-BRONCHI), including 4 RCTs and 386 patients suffered from non-CF bronchiectasis, found no significant differences in key pulmonary outcomes (mean FEV1, FVC, and annual pulmonary exarcebations) between hypertonic saline (HS) and non-hypertonic saline control group (Non-HS). Notably, our meta-analysis systematically evaluate subgroup on FEV1 outcome of long-term treatment of at least one year with frequent annual exarcebation rates remained consistent with the primary analysis.

Compared with previous meta-analyses [6, 7], our Hyper-BRONCHI metaanalysis provides a more rigorous and contemporary synthesis by incorporating the most recent evidence, including the CLEAR trial, and by conducting subgroup analyses restricted to low-risk-of-bias RCTs. This approach strengthens internal validity and allows a more precise assessment of the comparative efficacy of inhaled hypertonic saline versus other controls in non-cystic fibrosis bronchiectasis, independent of any adjuvant treatment effects.

Nonetheless, several limitations warrant consideration. The total sample size remained modest (193 randomized to HS and 193 to non-HS), which constrains statistical power, especially for annual exacerbation outcomes, which were reported in only two trials. Similarly, a recent study [12] of brensocatib, an oral, reversible inhibitor of dipeptidyl peptidase-1 (DPP-1), in patients with bronchiectasis required a large sample size; although the minimum estimated requirement was 1,000 participants, 1,721 patients were ultimately enrolled to demonstrate a modest reduction in exacerbation rates and a difference in FEV₁ decline. Some degree of heterogeneity is likely unavoidable given variations in HS osmolality, control interventions, and underlying patient comorbidities. Further, Despite differences in baseline severity metrics across trials, disease severity in the CLEAR trial [8] was assessed using the FACED score, with a reported median value of 2, corresponding to mild-to-moderate disease. In contrast, other included trials [3–5] primarily used FEV₁% predicted, with baseline values of values ≥ 60%, similarly reflecting moderate airflow limitation. Although FACED and FEV₁% predicted capture different dimensions of disease severity, both measures suggest broadly comparable baseline severity across studies. Nevertheless, these differences should be acknowledged as a potential source of heterogeneity in our analysis, and the pooled results should be interpreted with caution.

Although our search strategy was comprehensive, encompassing 780 records across three major databases, it remains possible that relevant studies located in other repositories were not captured. Quality of life outcomes were not predefined for analysis, and although two trials reported St George’s Respiratory Questionnaire scores [5, 8], methodological variability limits the ability to draw definitive conclusions. Notably, the CLEAR trial [8] demonstrated a mean difference of -0.3 (95% CI: -3.8 to 3.2), suggesting no clinically meaningful improvement in health-related quality of life with HS. Taken together, the current evidence indicates that while HS remains physiologically plausible and widely used, its clinical benefits in non-CF bronchiectasis are uncertain and warrant further high-quality investigation.

Overall, our meta-analysis found that inhaled HS did not produce significant improvement in FEV₁, FVC, or annual exacerbation frequency within one year in adults with non-CF bronchiectasis. These findings may reflect fundamental pathophysiological differences. In non-CF bronchiectasis, impaired mucociliary clearance initiates a self-perpetuating cycle of chronic infection, inflammation, and airway damage [13]. In contrast, CF bronchiectasis originates primarily from CFTR dysfunction, with dehydrated mucus, and abnormal airway surface hydration preceding inflammatory injury [14, 15]. Consequently, inhaled hypertonic saline, by hydrating the airway lumen, mitigating nasal potential difference and reducing mucus viscosity, appears to target CF-specific mechanisms more directly than those driving non-CF bronchiectasis.

A key finding of our HYPER-BRONCHI meta-analysis was the low certainty of evidence for mean FEV₁ and mean FVC, and very low certainty for annual pulmonary exacerbations. According to the GRADE framework, lung function outcomes were downgraded two levels due to inconsistency and risk of bias, reflecting methodological shortcomings across included trials. Specifically, Kellett and Roberts et al. (2011) was deemed high risk of bias due to inadequate blinding, missing outcome data related to tolerability, and insufficient reporting of allocation concealment and prespecified analyses, which likely contributed to heterogeneity and affected pooled estimates. The interpretation of pulmonary exacerbations was limited by heterogeneity in outcome definitions and reporting, necessitating analysis as continuous variables using standardized mean differences rather than rate-based measures. This limitation, alongside small sample sizes and wide confidence intervals, resulted in serious imprecision and very low certainty of evidence for this outcome.

Furthermore, as only four trials were included in the HYPER-BRONCHI meta-analysis, our ability to assess publication bias was limited, given the unreliability of both Egger’s test and funnel plots with such small numbers of studies. Similarly, sensitivity analyses using a leave-one-out approach were not feasible for the annual pulmonary exacerbations outcome.

Overall, these factors diminish confidence in the pooled estimates, suggesting that true effects may differ substantially from observed results. These findings emphasize the need for well-designed, adequately powered randomized controlled trials to evaluate hypertonic saline in non–CF bronchiectasis.

## Conclusion

In conclusion, the use of inhaled hypertonic saline in adults with non-CF bronchiectasis warrants further investigation through larger, high-quality RCTs with sufficient power to detect clinically meaningful improvements in pulmonary function, and importantly, reductions in exacerbation rates. Current evidence from the Hyper-BRONCHI analysis indicates that inhaled hypertonic saline may not produce substantial improvements in FEV₁ or FVC, underscoring the need for more robust studies to define its therapeutic role.

## Supplementary Information

Supplementary Material 1.

## Data Availability

All data generated or analysed during this study are found in following published Randomized Controlled Trials ( **REFERENCES** **^2^, ^16^, ^3^, ^4^** ). Supporting document ( **PRISMA checklist, GRADE table, RoB 2 table, and Table 1** ) used in the current study are available from the corresponding author upon reasonable request.
