# Supplementary material for: Hypertonic saline in non–cystic fibrosis bronchiectasis (Hyper-BRONCHI): an updated systematic review and meta-analysis
Source: BMC Pulm Med. 2026 Feb 12;26:122. doi: 10.1186/s12890-026-04176-4 (PMC12997658; doi:10.1186/s12890-026-04176-4)
Supplement: Supplementary file 1 — Supplementary Material 1. [file 12890_2026_4176_MOESM1_ESM.docx]

**Hyper-BRONCHI Study**

***Supporting documents***

**Search Strategy:**

("Hypertonic Saline Solution"[Mesh] OR "hypertonic saline" OR "hypertonic solution" OR "3% NaCl" OR "3% saline" OR "sodium chloride hypertonic")

AND

("Bronchiectasis"[Mesh] OR bronchiectasis OR "non-cystic fibrosis bronchiectasis" OR "non cystic fibrosis bronchiectasis" OR "muco-obstructive pulmonary disease" OR "non-CF bronchiectasis").

**Figure 1. Flowchart of study selection**.

**Identification of new studies via databases and registers**

Records identified from:

Databases (n = 780)

PubMed (n = 93)

Embase (n = 618)

Cochrane (n = 69)

Records removed *before screening*:

Duplicate records removed (n = 155)

**Identification**

Reports sought for retrieval

(n = 10)

Records screened

(n = 625)

Records excluded by title and abstract

(n = 615)

Reports not retrieved

(n = 0)

**Screening**

Reports excluded:

Abstract conference (n = 1)

Observational Studies (n = 1)

Reported in Chinese (n = 1)

Single-armed RCT (n = 1)

Only combined hypertonic Saline and hyaluronic acid (n = 1)

Adjuvant therapy only (n = 1)

Reports assessed for eligibility

(n = 10)

Our main analysis:

- Total studies included in review

(n = 4)

- Reports of total included studies

(n = 4; 4 RCTs)

**Included**

*From:*  Page MJ, McKenzie JE, Bossuyt PM, Boutron I, Hoffmann TC, Mulrow CD, et al. The PRISMA 2020 statement: an updated guideline for reporting systematic reviews. BMJ 2021;372:n71. doi: 10.1136/bmj.n71

Table 1. Baseline characteristics of included studies

| Study | Design | Group | Age, mean (SD) | Sex (M/F) | BMI (kg/m²) | FVC % predicted (SD) | FEV₁ % predicted (SD) | Annual exacerbations | Key inclusion criteria |
| --- | --- | --- | --- | --- | --- | --- | --- | --- | --- |
| Kellett & Robert, 2011 [9] | Cross-over | — | 56.6 (14.6) | 14 / 14 | N/R | 77.8 (23.4) | 66.4 (26.1) | 2.6 | >18 yrs; HRCT-confirmed bronchiectasis ≤4 years; stable chronic treatment allowed. |
| Nicolson et al., 2012 [10] | Parallel RCT | HTS | 58  (15) | 7 / 13 | 27.9 | 98.5 (17.8) | 84.8 (20.5) | 5.0 (median) | >18 yrs; HRCT-confirmed bronchiectasis; clinically stable; daily sputum; ≥2 exacerbations/yr (past 2 yrs) |
|  |  | IS | 56  (15) | 8 / 12 | 28.2 | 97.1 (18.0) | 80.4 (21.1) | 5.5 (median) |  |
| Herrero-Cortina et al.,  2018 [8] | Cross-over | — | 64.0 (17.5) | 10 / 18 | 24.3 | NR | 60.9 (24.6) | NR | >18 yrs; HRCT-confirmed bronchiectasis; stable ≥4 weeks; sputum ≥10 g/24h; able to perform ACT. |
| Bradley et al., 2025 [3] | Parallel RCT | HTS | 65.3 (13.6) | 60 / 86 | 28.1 | NR | NR | 3.4 ± 2.1 | ≥18 yrs; CT-confirmed bronchiectasis; ≥2 exacerbations/yr (or ≥1 post-COVID-19); daily sputum. |
|  |  | No HTS | 66.1 (12.3) | 57 / 85 | 27.6 | NR | NR | 3.6 ± 2.1 |  |

Data are presented as mean ±SD unless otherwise specified. “Median” indicates median (IQR). NR = not reported. HRCT = high-resolution computed tomography; ACT = airway clearance technique; HTS = hypertonic saline; IS = isotonic saline.

**Sensitivity analysis, sequentially removing individual trials to assess their influence on the overall results**

**Supplementary Figure 3.1 – 3.4, Mean FEV1:**

**3.1**

**
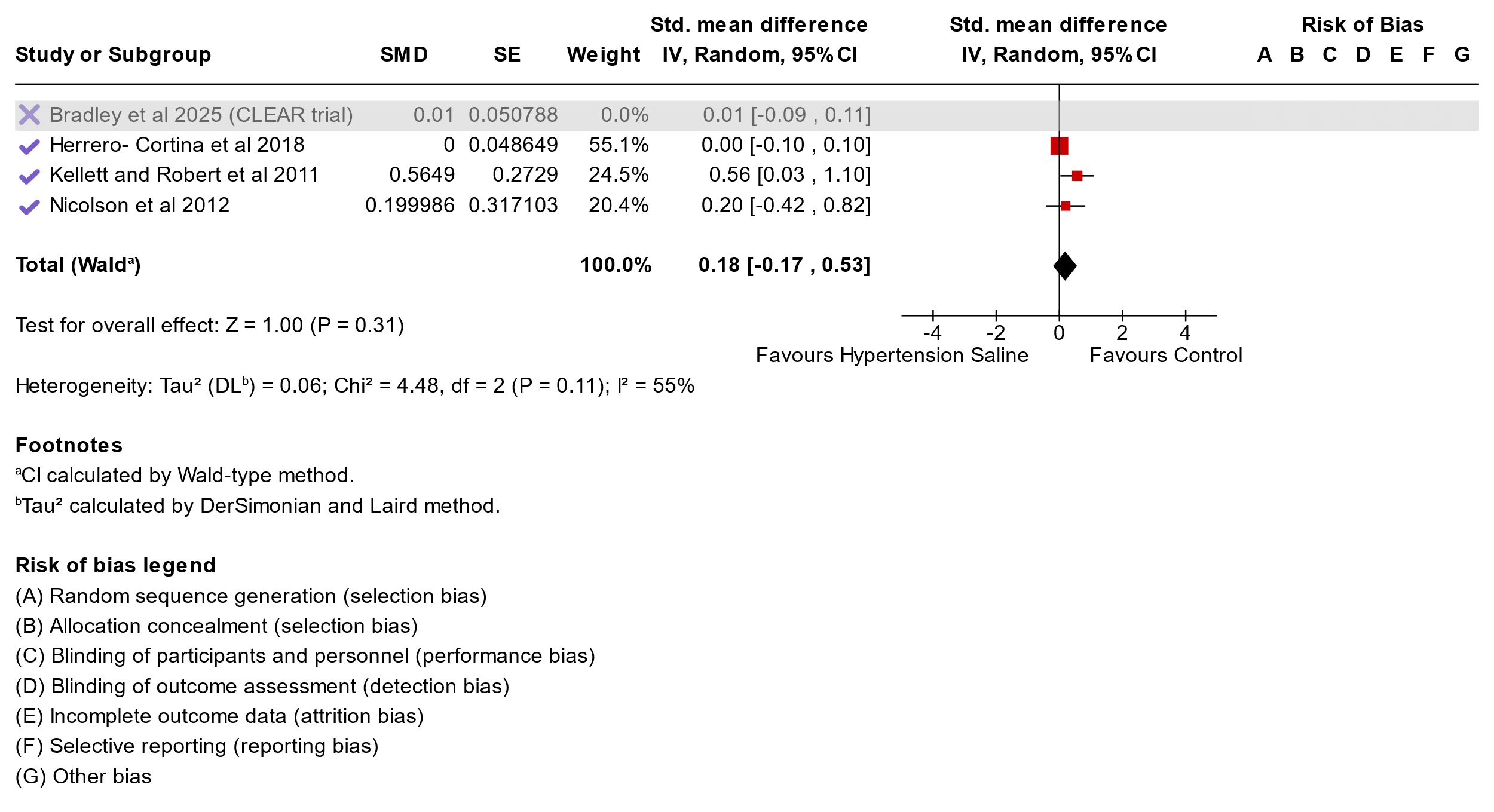
**

**3.2**

**
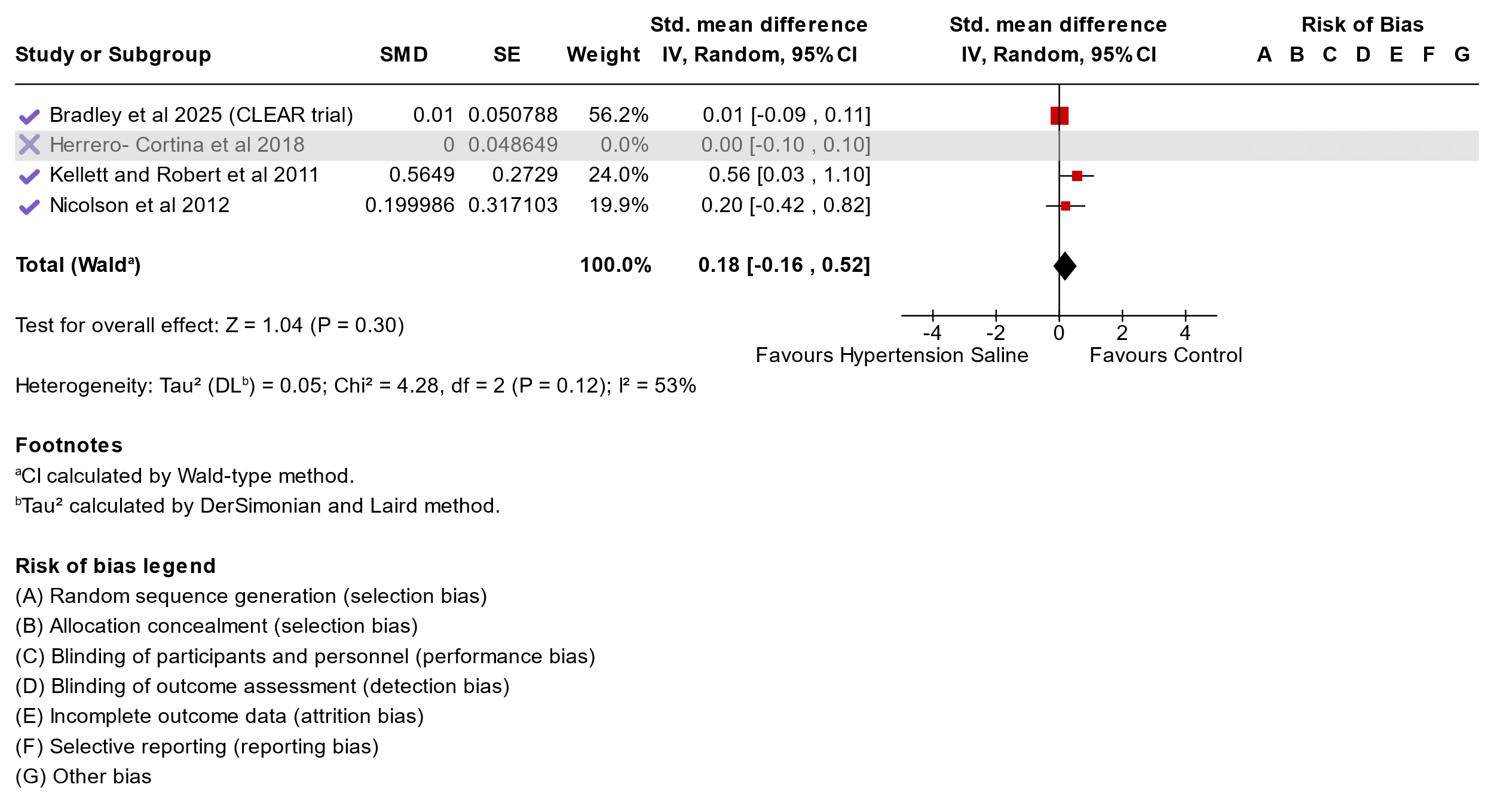
**

**3.3**

**
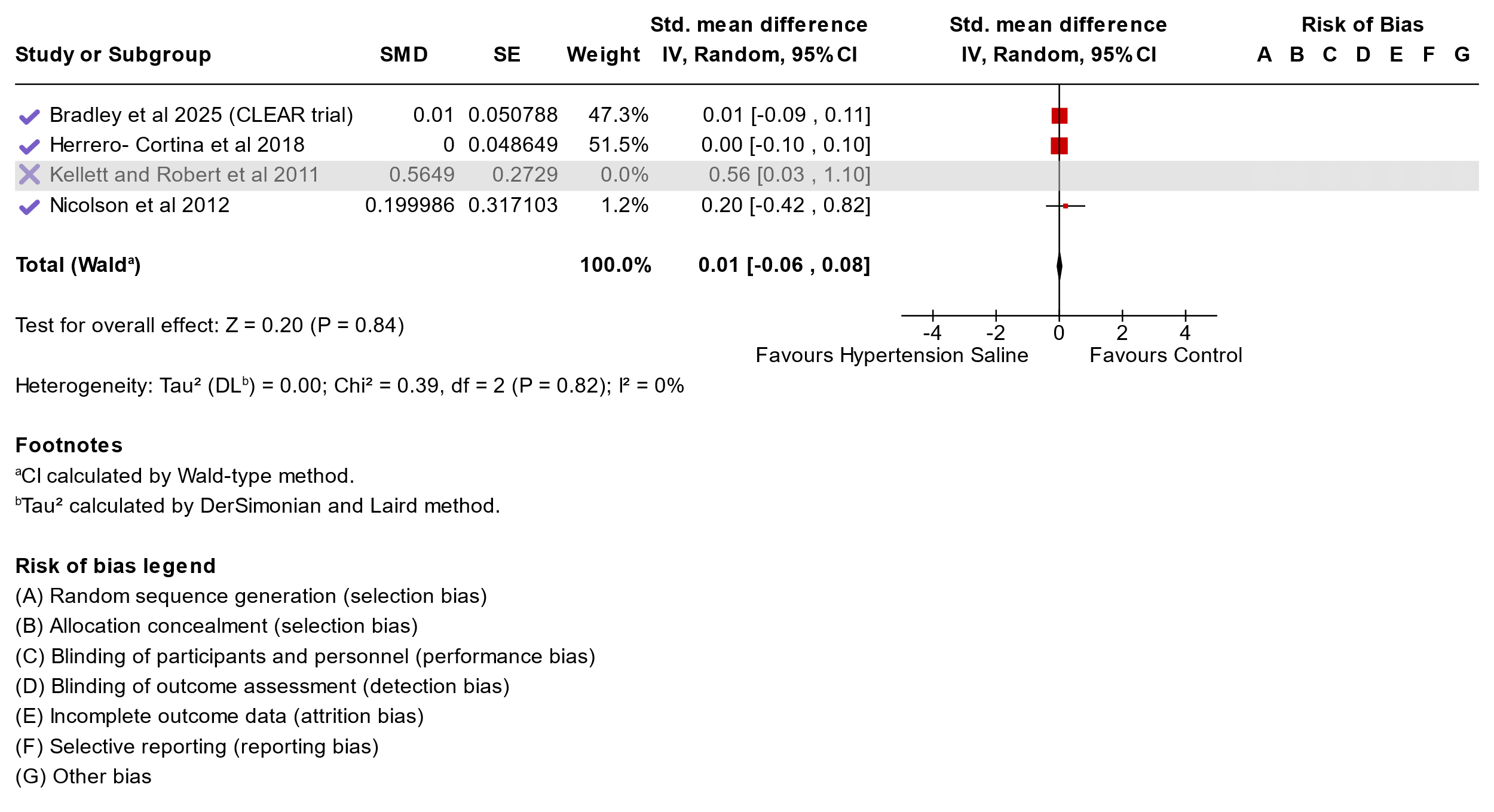
**

**3.4**

**
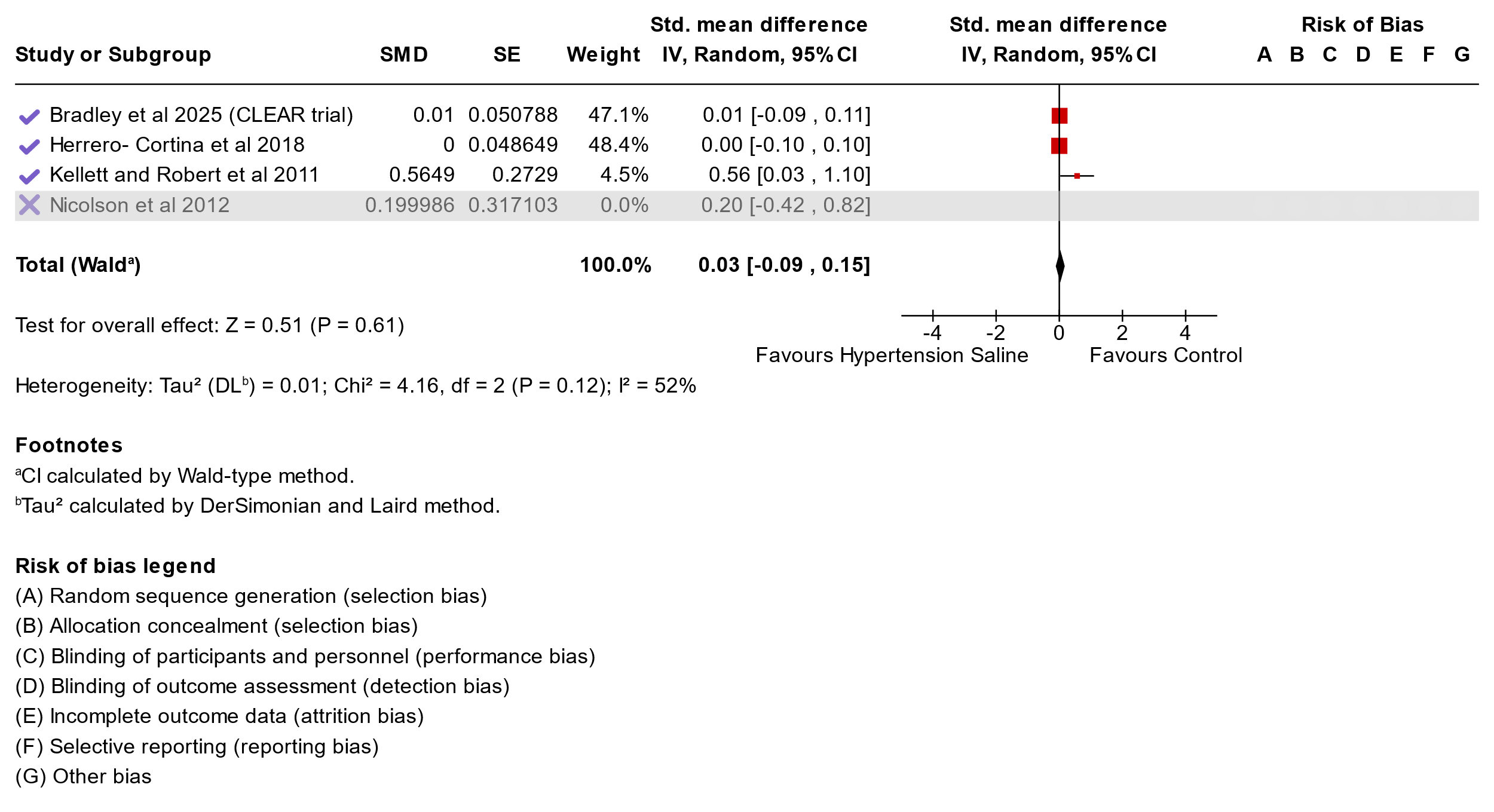
**

**Supplementary Figure 4.1 – 4.4, Mean FVC:**

**4.1**

**
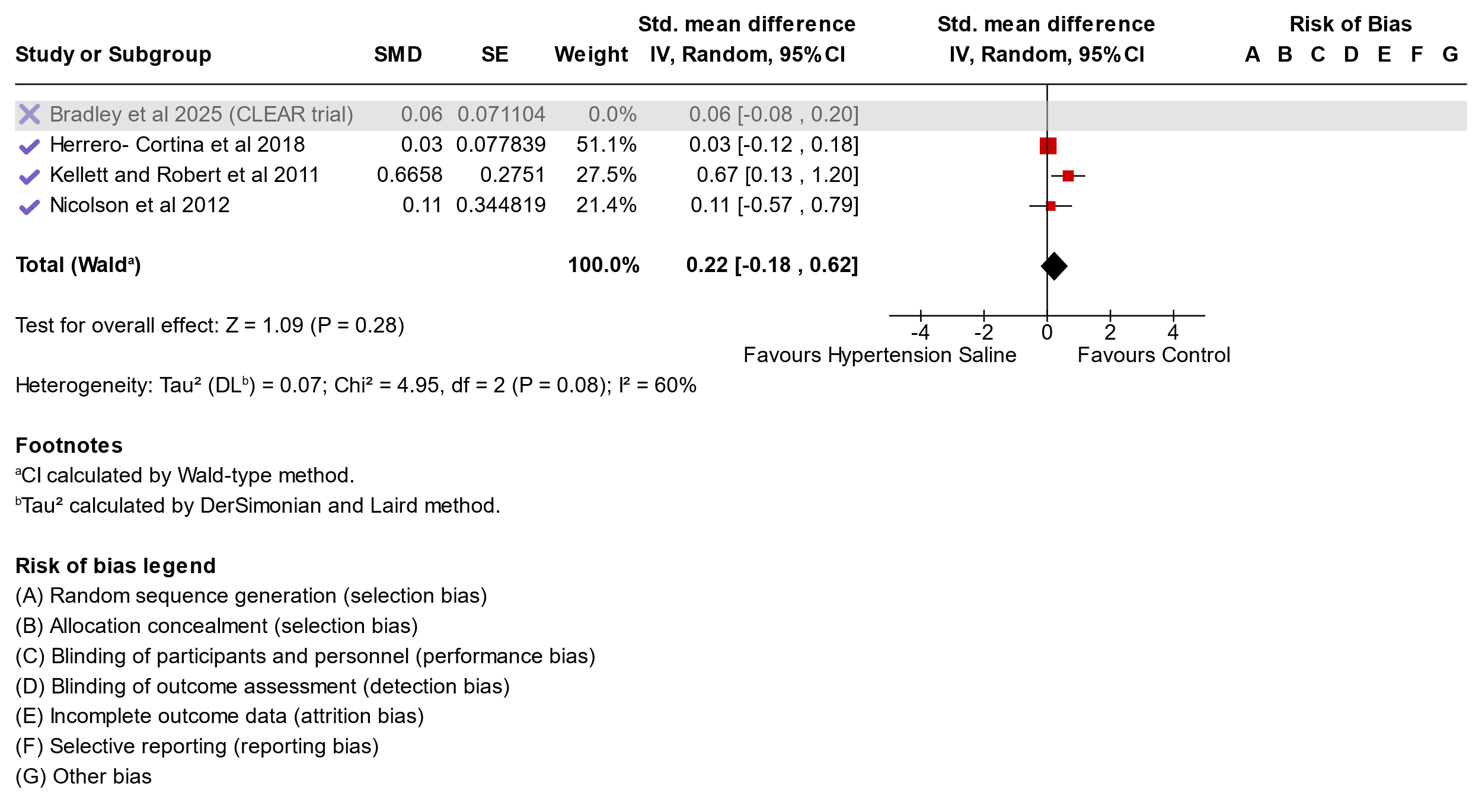
**

**4.2**

**
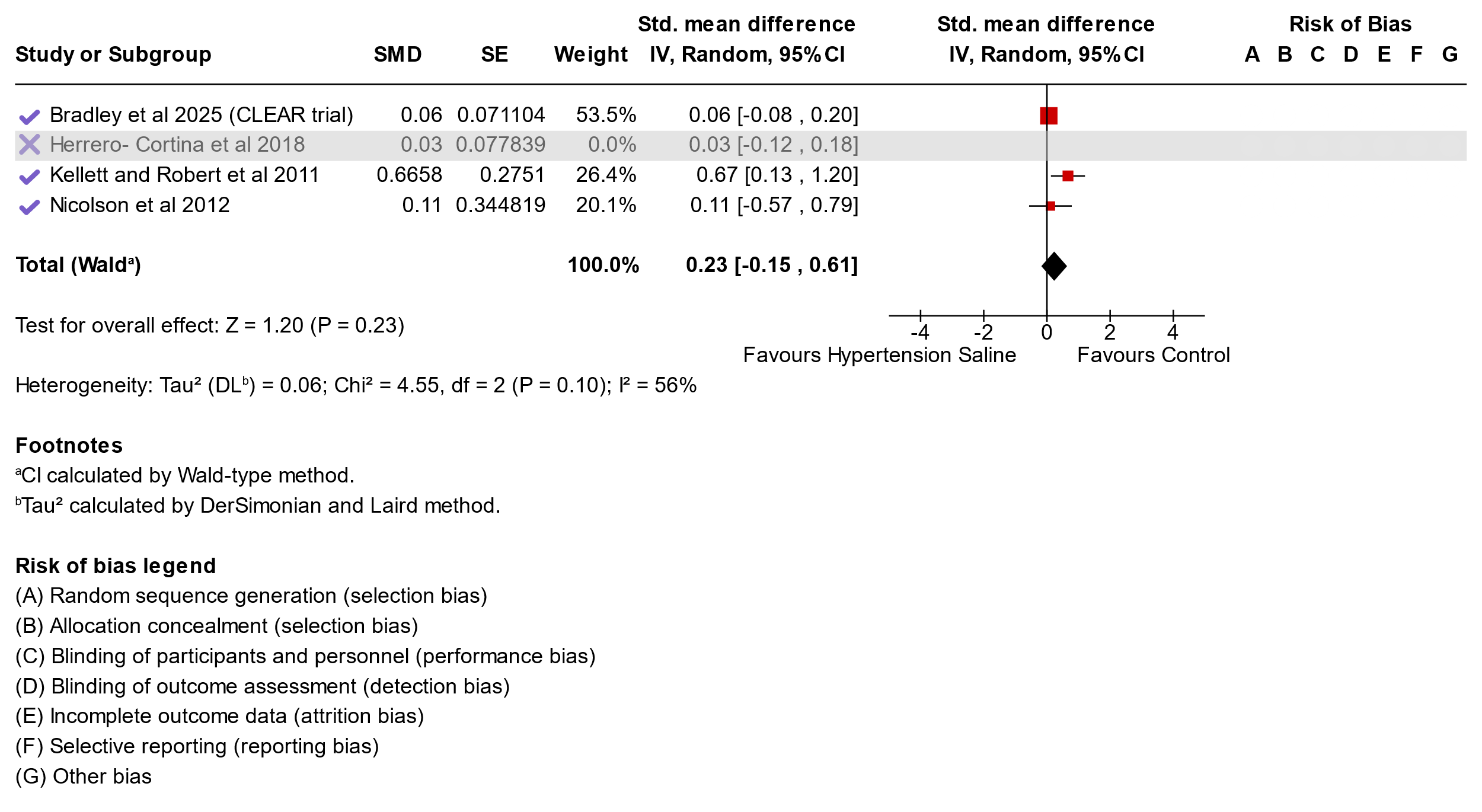
**

**4.3**

**
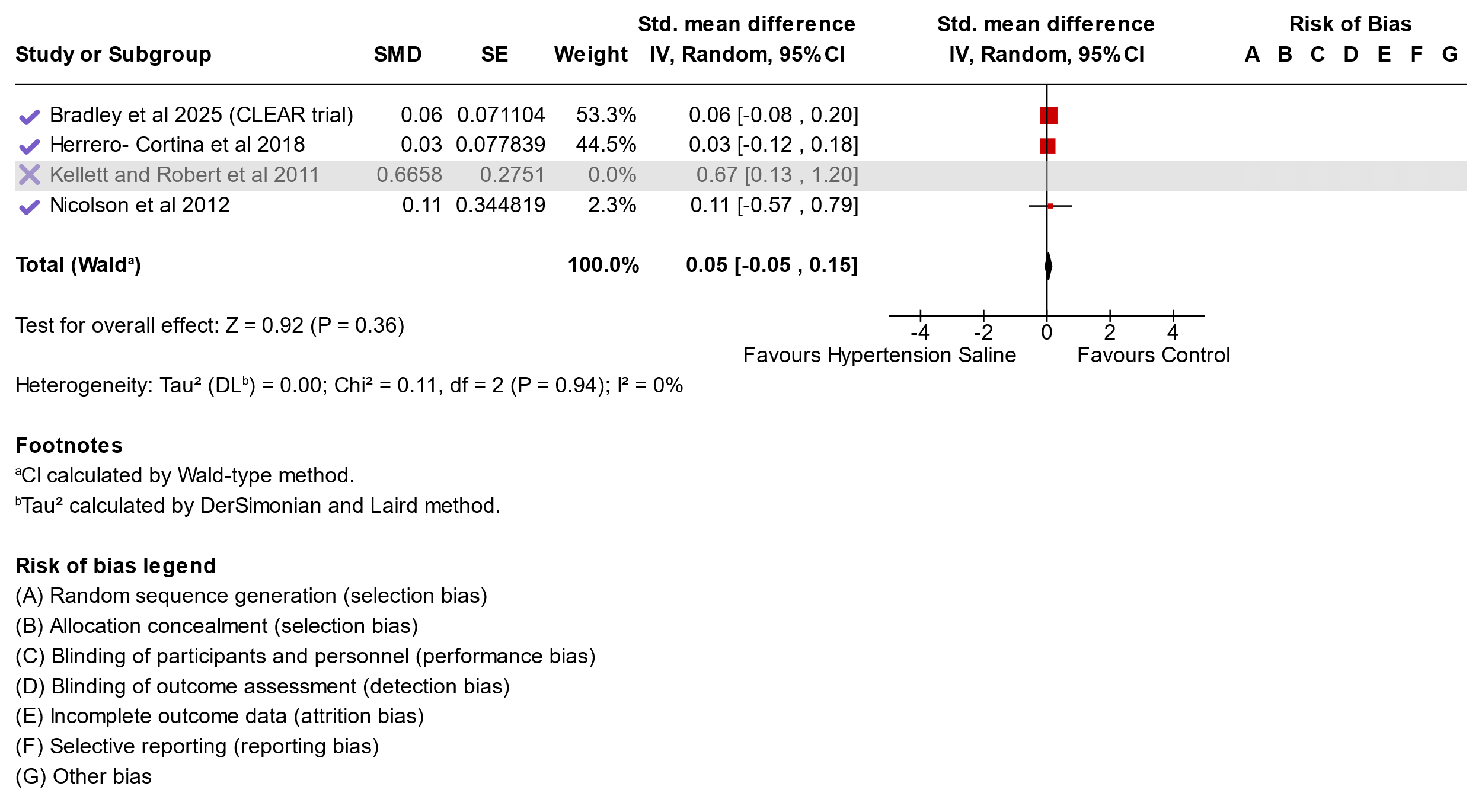
**

**4.4**

**
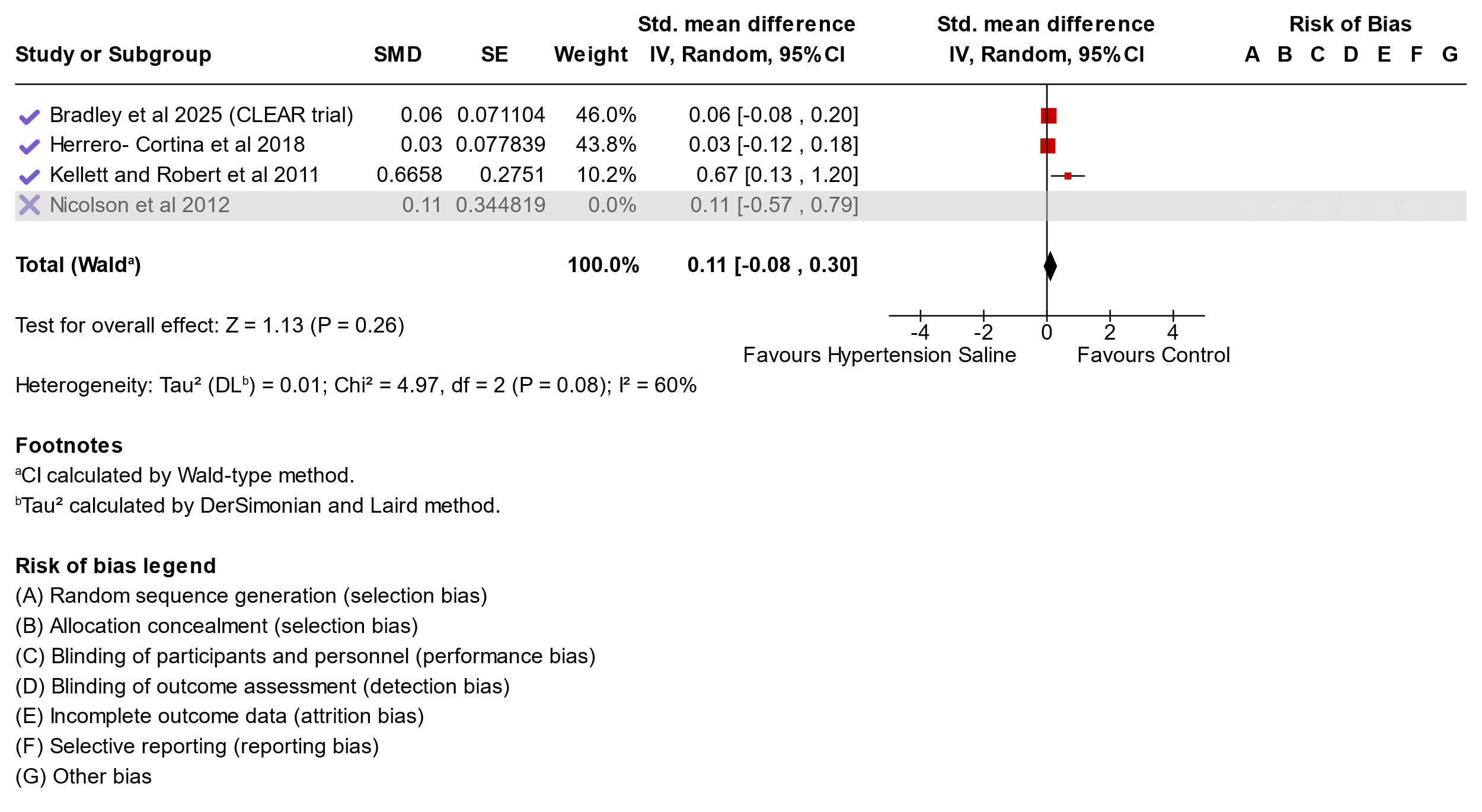
**

**Table 2.** **Risk of bias summary for randomized studies (RoB 2)**

| **Study** | **Bias from randomization process** | **Bias due to deviations from intended interventions** | **Bias due to missing outcome data** | **Bias in measurement of the outcomes** | **Bias in selection of the reported result** | **Overall risk of bias** |
| --- | --- | --- | --- | --- | --- | --- |
| Bradly et al. 2025 (CLEAR) | Low | Low | Low | Low | Low | Low |
| Herrero-Cortina et al. 2018 | Low | Low | Some  Concerns | Low | Low | Some  Concerns |
| Nicolson et al. 2012 | Low | Low | Low | Low | Low | Low |
| Kellett and Robert et al. 2011 | Low | Some  Concerns | Some  Concerns | Low | Some concerns | High |

Table 2 summarizes the risk of bias assessments for the included studies. Two of the four trials were judged to have a low risk of bias. The trial by Herrero-Cortina et al. (2018) was rated as having “some concerns” due to missing outcome data—only 23 of 28 participants completed the study, with 5 participants (18%) withdrawing mainly because of time burden or tolerability issues. The study by Kellett and Roberts et al. (2011) was assessed as having a high risk of bias, driven by “some concerns” across three domains: insufficient blinding (single blinding only), missing data related to tolerability, and limited reporting on allocation concealment and prespecified analyses.

| **Hypertonic Saline (HS) compared to Non-Hypertonic solution (Non-HS) for non-cystic firbosis Bronchiectasis (GRADE Table)** | | | | | |
| --- | --- | --- | --- | --- | --- |
| **Patient or population:** Non-cystic Bronchiectasis  **Setting:** Inpatient and Outpatient  **Intervention:** Hypertonic Saline (HS)  **Comparison:** Non-Hypertonic (Non-HS). | | | | | |
| **Outcomes** | **№ of participants (studies) Follow-up** | **Certainty of the evidence (GRADE)** | **Relative effect (95% CI)** | **Anticipated absolute effects** | |
|  |  |  |  | **Risk with HS.** | **Risk difference with Non-HS** |
| Mean FEV1 assessed with: Standard Mean Difference | 363  (4 RCTs) | ⨁⨁◯◯ Low^d,a^ | - | - | SMD **0.03 SD higher** (0.07 lower to 0.13 higher) |
| Mean FVC assessed with: Standard Mean Difference | 363  (4 RCTs) | ⨁⨁◯◯ Low^d,a^ | - | - | SMD **0.1 SD higher** (0.06 lower to 0.25 higher) |
| Mean number of pulmonary exarcebations assessed with: Standard Mean Difference | 307  (2 RCTs) | ⨁◯◯◯  Very Low^a,b,d^ | - | - | SMD **0.03 exacerbations higher** (1.08 lower to 1.14 higher) |

| **GRADE Working Group grades of evidence High certainty: we are very confident that the true effect lies close to that of the estimate of the effect. Moderate certainty: we are moderately confident in the effect estimate: the true effect is likely to be close to the estimate of the effect, but there is a possibility that it is substantially different. Low certainty: our confidence in the effect estimate is limited: the true effect may be substantially different from the estimate of the effect. Very low certainty: we have very little confidence in the effect estimate: the true effect is likely to be substantially different from the estimate of effect.** |
| --- |

**Explanations**

**a. Inconsistency was judged by forest plot inspection, I², and Chi² rather than I² cut-offs**

**b. Imprecision was rated down when 95% CIs crossed narrow clinical thresholds of importance.**

**c. Publication bias was assessed using Egger’s test; if p < 0.05, certainty of evidence was downgraded for risk of publication bias.**

**d. Risk of bias: downgraded for deviations from intended interventions, and from missing data on outcomes.**

| **Section and Topic** | **Item #** | **Checklist item** | **Paragraph /Page where item is reported** |
| --- | --- | --- | --- |
| **TITLE** | | |  |
| Title | 1 | Identify the report as a systematic review. | 1 (Page) |
| **ABSTRACT** | | |  |
| Abstract | 2 | See the PRISMA 2020 for Abstracts checklist. | N/A |
| **INTRODUCTION** | | |  |
| Rationale | 3 | Describe the rationale for the review in the context of existing knowledge. | Paragraph 1 |
| Objectives | 4 | Provide an explicit statement of the objective(s) or question(s) the review addresses. | Paragraph 1 |
| **METHODS** | | |  |
| Eligibility criteria | 5 | Specify the inclusion and exclusion criteria for the review and how studies were grouped for the syntheses. | Paragraph 2 |
| Information sources | 6 | Specify all databases, registers, websites, organisations, reference lists and other sources searched or consulted to identify studies. Specify the date when each source was last searched or consulted. | Paragraph  2 |
| Search strategy | 7 | Present the full search strategies for all databases, registers and websites, including any filters and limits used. | 8 |
| Selection process | 8 | Specify the methods used to decide whether a study met the inclusion criteria of the review, including how many reviewers screened each record and each report retrieved, whether they worked independently, and if applicable, details of automation tools used in the process. | Paragraph  3 |
| Data collection process | 9 | Specify the methods used to collect data from reports, including how many reviewers collected data from each report, whether they worked independently, any processes for obtaining or confirming data from study investigators, and if applicable, details of automation tools used in the process. | Paragraph  3 |
| Data items | 10a | List and define all outcomes for which data were sought. Specify whether all results that were compatible with each outcome domain in each study were sought (e.g. for all measures, time points, analyses), and if not, the methods used to decide which results to collect. | Paragarph  2 |
|  | 10b | List and define all other variables for which data were sought (e.g. participant and intervention characteristics, funding sources). Describe any assumptions made about any missing or unclear information. | N/A |
| Study risk of bias assessment | 11 | Specify the methods used to assess risk of bias in the included studies, including details of the tool(s) used, how many reviewers assessed each study and whether they worked independently, and if applicable, details of automation tools used in the process. | Paragraph  3 |
| Effect measures | 12 | Specify for each outcome the effect measure(s) (e.g. risk ratio, mean difference) used in the synthesis or presentation of results. | Paragraph  3 |
| Synthesis methods | 13a | Describe the processes used to decide which studies were eligible for each synthesis (e.g. tabulating the study intervention characteristics and comparing against the planned groups for each synthesis (item #5)). | N/A |
|  | 13b | Describe any methods required to prepare the data for presentation or synthesis, such as handling of missing summary statistics, or data conversions. | N/A |
|  | 13c | Describe any methods used to tabulate or visually display results of individual studies and syntheses. | N/A |
|  | 13d | Describe any methods used to synthesize results and provide a rationale for the choice(s). If meta-analysis was performed, describe the model(s), method(s) to identify the presence and extent of statistical heterogeneity, and software package(s) used. | Paragraph  3 |
|  | 13e | Describe any methods used to explore possible causes of heterogeneity among study results (e.g. subgroup analysis, meta-regression). | Paragraph |
|  | 13f | Describe any sensitivity analyses conducted to assess robustness of the synthesized results. | N/A |
| Reporting bias assessment | 14 | Describe any methods used to assess risk of bias due to missing results in a synthesis (arising from reporting biases). | Paragraph  3 |
| Certainty assessment | 15 | Describe any methods used to assess certainty (or confidence) in the body of evidence for an outcome. | Paragraph  3 |
| **RESULTS** | | |  |
| Study selection | 16a | Describe the results of the search and selection process, from the number of records identified in the search to the number of studies included in the review, ideally using a flow diagram. | Paragraph  4 |
|  | 16b | Cite studies that might appear to meet the inclusion criteria, but which were excluded, and explain why they were excluded. | N/A |
| Study characteristics | 17 | Cite each included study and present its characteristics. | Paragraph  4 |
| Risk of bias in studies | 18 | Present assessments of risk of bias for each included study. | Paragraph  6 |
| Results of individual studies | 19 | For all outcomes, present, for each study: (a) summary statistics for each group (where appropriate) and (b) an effect estimate and its precision (e.g. confidence/credible interval), ideally using structured tables or plots. | Paragraph  6 |
| Results of syntheses | 20a | For each synthesis, briefly summarise the characteristics and risk of bias among contributing studies. | N/A |
|  | 20b | Present results of all statistical syntheses conducted. If meta-analysis was done, present for each the summary estimate and its precision (e.g. confidence/credible interval) and measures of statistical heterogeneity. If comparing groups, describe the direction of the effect. | Paragraph  5 |
|  | 20c | Present results of all investigations of possible causes of heterogeneity among study results. | Paragraph  6 |
|  | 20d | Present results of all sensitivity analyses conducted to assess the robustness of the synthesized results. | N/A |
| Reporting biases | 21 | Present assessments of risk of bias due to missing results (arising from reporting biases) for each synthesis assessed. | Paragraph  6 |
| Certainty of evidence | 22 | Present assessments of certainty (or confidence) in the body of evidence for each outcome assessed. | Paragraph  5 |
| **DISCUSSION** | | |  |
| Discussion | 23a | Provide a general interpretation of the results in the context of other evidence. | Paragraph  8 |
|  | 23b | Discuss any limitations of the evidence included in the review. | Paragraph 7 |
|  | 23c | Discuss any limitations of the review processes used. | Paragraph 7 |
|  | 23d | Discuss implications of the results for practice, policy, and future research. | Paragraph |
| **OTHER INFORMATION** | | |  |
| Registration and protocol | 24a | Provide registration information for the review, including register name and registration number, or state that the review was not registered. | Paragraph  2 |
|  | 24b | Indicate where the review protocol can be accessed, or state that a protocol was not prepared. | N/A |
|  | 24c | Describe and explain any amendments to information provided at registration or in the protocol. | N/A |
| Support | 25 | Describe sources of financial or non-financial support for the review, and the role of the funders or sponsors in the review. | Page  5 |
| Competing interests | 26 | Declare any competing interests of review authors. | Page  5 |
| Availability of data, code and other materials | 27 | Report which of the following are publicly available and where they can be found: template data collection forms; data extracted from included studies; data used for all analyses; analytic code; any other materials used in the review. | Page  5 |

*From:*  Page MJ, McKenzie JE, Bossuyt PM, Boutron I, Hoffmann TC, Mulrow CD, et al. The PRISMA 2020 statement: an updated guideline for reporting systematic reviews. BMJ 2021;372:n71. doi: 10.1136/bmj.n71. This work is licensed under CC BY 4.0. To view a copy of this license, visit <https://creativecommons.org/licenses/by/4.0/>
